# Nutritional quality modulates trait variability

**DOI:** 10.1186/s12983-018-0297-2

**Published:** 2018-12-05

**Authors:** Adrian Brückner, Romina Schuster, Katja Wehner, Michael Heethoff

**Affiliations:** 1 Ecological Networks, Technische Universität Darmstadt, Schnittspahnstraße 3, 64287 Darmstadt, Germany; 2 Division of Biology and Biological Engineering, California Institute of Technology, 1200 E California Boulevard, Pasadena, CA 91125 USA

**Keywords:** Trait plasticity, Functional traits, Parthenogenesis, Nutritional ecology, Nutritional balance, Threshold elemental ratio, Oribatid mites, *Archegozetes longisetosus*

## Abstract

**Background:**

Trait based functional and community ecology is *en vogue*. Most studies, however, ignore phenotypical diversity by characterizing entire species considering only trait means rather than their variability. Phenotypical variability may arise from genotypical differences or from ecological factors (e.g., nutritionally imbalanced diet), and these causes can usually not be separated in natural populations. We used a single genotype from a parthenogenetic model system (the oribatid mite *Archegozetes longisetosus* Aoki) to exclude genotypical differences. We investigated patterns of dietary (10 different food treatments) induced trait variation by measuring the response of nine different traits (relating to life history, morphology or exocrine gland chemistry).

**Results:**

Nutritional quality (approximated by carbon-to-nitrogen ratios) influenced all trait means and their variation. Some traits were more prone to variation than others. Furthermore, the “threshold elemental ratio”- rule of element stoichiometry applied to phenotypic trait variation. Imbalanced food (i.e. food not able to fully meet the nutritional demands of an animal) led to lower trait mean values, but also to a higher variation of traits.

**Conclusion:**

Imbalanced food led not only to lower trait value averages, but also to higher trait variability. There was a negative relationship between both parameters, indicating a direct link of both, average trait levels and trait variation to nutritional quality. Hence, variation of trait means may be a predictor for general food quality, and further indicate trade-offs in specific traits an animal must deal with while feeding on imbalanced diets.

**Electronic supplementary material:**

The online version of this article (10.1186/s12983-018-0297-2) contains supplementary material, which is available to authorized users.

## Background

The theory of phenotypic plasticity predicts that virtually all biological systems inherently include variation of characters at all organization levels [1–3]. This variation can be induced by virtually any external factor, leading to a nearly infinite number of possible phenotypic specificities [4, 5]. Phenotypic plasticity can therefore be defined as the degree of a single genotype to express variation in multiple traits leading to different phenotypes in response to environmental gradients [3, 6]. Trait based approaches are *en vogue* in all fields of ecology [7, 8], and it has been assumed that trait means of a species can be used to sufficiently characterize communities and affiliated functionality [9, 10]. Natural populations, however, consists of phenotypically diverse individuals, possessing variable traits [3, 11, 12]. Furthermore, trait variability of individuals within populations is essential for evolutionary processes via natural selection, as the latter acts on the individuals’ phenotypes influenced by a given ecological setting [1, 6, 11]. That is why understanding causes and mechanism leading to such trait variation of individuals can give unprecedented resolution to explain evolutionary ecology dynamics underpinning community structure, trait assemblages, and stability (e.g., [11, 13, 14]).

Theory [15], but also some experimental evidence (e.g., [16, 17]) suggest that phenotypic plasticity may arises from trade-offs among multiple traits within a species that are ultimately caused by imbalances in elemental stoichiometry or nutritional components of food. For instance, nutrients influence fitness related traits (e.g., [18–21]), but also body shapes [22], or intraspecific chemical communication (e.g. [23]) of an animal, and may even alter biological rules [24]. In this context, a resource can be considered optimal if i) it enables an animal to respond with the highest possible trait mean (= balanced diet hypothesis; [25]), but also ii) can sustain phenotypes with low trait variability and thus a stable performance (see [18, 20, 26]). For instance, two recent meta-analyses suggest that mixed-, rather than single-food diets result in significantly higher average fitness [27] and reduce intraspecific variance in fitness [28], because mixed foods are more likely to represent an optimal, balanced diet close to an animals intake target [25]. Bunning et al. [29] as well as Han & Dingemanse [30] tested these hypotheses in the context of reproductive performance and behavioral stability, respectively, and found that optimal dietary choice indeed led to an improved average expression and a reduced variance of their selected traits. Still, reaction norms of only a relatively limited number of traits towards a broad gradient of macronutrients [20] or elements [31] have been tested for a few species and the relationship of means, variation and elemental balance across multiple traits of different types (e.g. adaptive defense, morphological or reproductive traits) has, to our best knowledge, not been investigated in a single species under genotypic control.

Here, we harness the power of a unique model system – the all-female parthenogenetic and mono-genotypic oribatid mite *Archegozetes longisetosus* ran lineage, which reproduces via automixis with an inverted meiosis resulting in clonal offspring [32, 33]. While heritable genetic variation of traits cannot clearly be distinguished from environmentally induced trait variation (phenotypic plasticity) in sexual species, clonal systems provide the possibility to quantify reaction norms of traits and their variation (e.g., [34–37]). We selected a variety of different individual and demographic life-history, morphological and defensive traits to quantify their response to food with different nutritional quality. We fed ten semi-natural resources, differing in C/N ratios, to the mites (all are descendants from a single mother and share an identical genotype), and measured the mean and variation of traits along this food quality gradient. Thus, we exploit our “phenotypically-tractable” model species (i.e. a model system without genotypic, but only phenotypic variation), to decipher patterns and mechanisms of dietary induced trait plasticity. More specifically we ask how nutritional quality (approximated by C/N ratios) influences the expression of multiple traits regarding i) trait means, ii) trait variation, and iii) differences among traits.

Based on recent meta-analyses [27, 28] about the effect of diet mixing, we expect similar patterns for the semi-natural single diets; mites which fed on resources with a balanced C/N-ratio (defined as a ratio equal or close to the mites’ stoichiometrical demands) should express a higher average and a lower variance across multiple traits. As no intake target (i.e. species specific optimal elemental/nutritional composition of food) is known for any mite species or soil decomposer it is not possible to predict an optimal C/N intake ratio. However, even the effects of single diets on our phenotypically tractable model species should be strong, as the clonal reproduction eliminates genotypic variation. Hence, we also expect to find a direct negative relationship of the expressed means and variances of traits across resources, which would further support the balanced diet hypothesis [20, 25].

## Materials and methods

### Experimental setup and trait selection

*Archegozetes longesitosus* ran [32] were reared at approx. 28 °C and 80–85% relative humidity in constant darkness on one out of ten resources for several generations (approx. 18 month, corresponding to seven to twelve generations depending on the food). All specimens of this strain are genotypically identical [32]. The ten resources (all applied as dried powders) were blood meal (blood; Common Baits, Rosenfeld, Germany), bone meal (bone; Canina Pharma GmbH, Hamm, Germany), *Spirulina* powder (spirulina; Interaquaristik, Biedenkopf-Breidenstein, Germany), shiitake fungus powder (fungi; Arche Naturprodukte GmbH, Hilden, Germany), grinded dry yeast (yeast; Rapunzel Naturkost GmbH, Legau, Germany), *Chlorella* powder (chlorella; Naturya, Bath, UK), hemp protein powder (hemp; Naturya, Bath, UK), sweet lupine flour (lupine; Govinda Natur GmbH, Neuhofen, Germany), grinded mixed pollen grain (pollen; Ascopharm GmbH, Wernigerode, Germany) and wheat grass powder (wheat; Naturya, Bath, UK). A detailed list of macro-elemental and nutritional composition can be found in the Additional file 1. Food and water were provided ad libitum three times a week. For each resource, specimens were cultured in three separated plastic boxes (100x100x50 mm) grounded with 2 cm mixture of plaster of Paris/activated charcoal mixture (9:1).

**Fig. 1:**
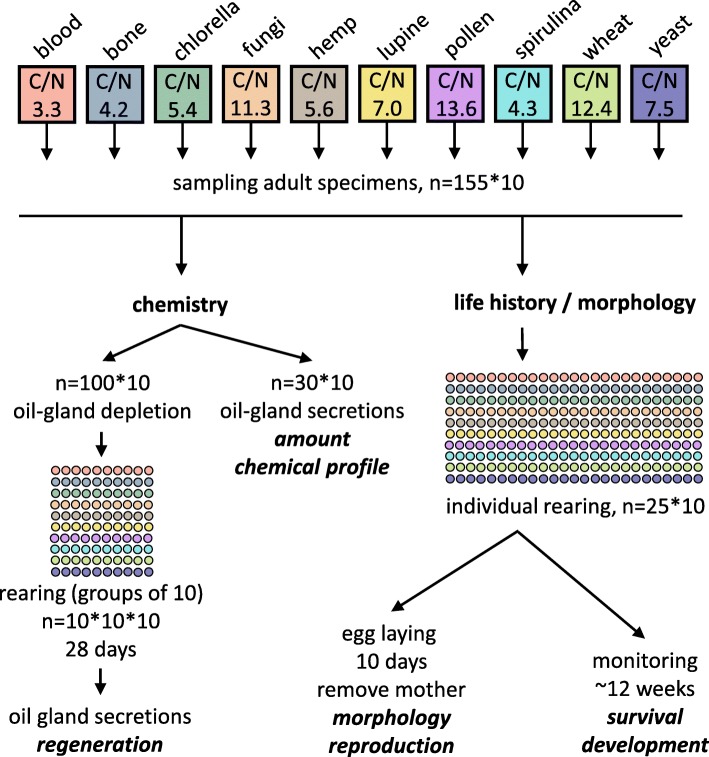
Conceptual scheme depicting the experimental design of this study. Color code corresponds to the ten resources and is used throughout the study. Circles represent sample replicates; sample sizes are also denoted in the figure

As traits we selected life-history, morphological and chemical characters (Fig. 1, Table 1). The life-history traits were selected to quantify the reproductive fitness of one female based on its offspring [35, 37, 38], while morphological traits were used to describe changes in size and body shape [2]. All chemical traits were related to defensive exocrine opisthonotal oil-glands [39, 40]. Oil-gland chemistry is assumed to be highly adaptive because it plays an important role in structuring feeding interactions in soil food webs and enables oribatid mites to live in an “enemy-free space” [41, 42], i.e. a conceptual way of living that reduces or eliminate a species’ vulnerability against predators [43].

**Table 1:** Overview of the nine selected traits of *Archegozetes longisetosus* measured in this study, as well as a definition of each character and general descriptions of these traits in a general ecological context

	Trait definition (this study)	Trait description	References
Chemistry			
Amount	Amount of defensive secretions of one individual, standardized by its dry weight (ng/μg)	In reservoir based chemical defense the amount is a primary factor to predict how often an animal is able to defend itself against predators and competitors	[66, 67]
Composition	Relative composition (%) of the defensive secretion of one individual	The composition of a defensive chemical blend can determine its effectivity against predators, but can also be a consequence of physiological changes/stress of an individual	[66, 68, 69]
Regeneration	Percentage of individuals (%) per group (*n* = 10) which regenerated their defensive secretions over time	The regeneration of defensive secretion is essential to be defended against predators at all, but also to understand the costs of secretion regeneration	[45, 70, 71]
Life-history			
Developmental time	Weighted arithmetic mean of developmental time of one females’ offspring	The first three life-history parameters (developmental time, survival and number of offspring) describe the reproductive fitness	[20, 35, 72]
Survival	Percentage (%) of surviving offspring of one female based on the counted number of laid eggs and newly enclosed adults		
Offspring	Counted number of surviving offspring of one female		
Output	Dry weight of one females’ entire offspring	The biomass output may also describe the fitness, but also quantifies the ability of a mother to translocate biomass from the resource to her offspring	[73, 74]
Morphology			
Body mass	Dry weight of the initially used females (mothers)	Body mass is a universal predictor of many ecological processes (e.g., metabolism, abundance, or predation)	[75–77]
Morphometry	Eleven morphometric characters of the initially used females (for details see Additional file 2)	The shape of an individual plays an important role in basic physiological processes, but also influences predation (by altered handling approaches by predators) and may be used to predict other characters	[22, 78, 79]

At the start of the experiment we selected young (approximately one week after eclosing) adult individuals from their original culture-plates. We directly used 130 specimens per resource (= 1300 in total) for chemical experiments, while 25 specimens per resource (= 250 in total) were individually redistributed into smaller culture boxes (45 × 40 × 35 mm; grounded with the plaster of Paris mixture) for further experiments.

#### Chemical experiments and analysis

Oil-gland extractions of 30 specimens from each of the resources (*n* = 10*30) were accomplished by immersing individuals in 50 μl hexane (GC grade, 98% purity purchased from Merck, Darmstadt, Germany) for 3 min (see Fig. 1). The extraction solvent also contained tetradecane (1 ng/μl; ≥99.8%, analytical standard, purchased from Sigma-Aldrich, Munich, Germany) as an internal standard. Crude extracts were used for gas chromatography-mass spectrometry (GC/MS) analyses and mite specimens were stored at − 20 °C for further measurements. Samples were analyzed with a QP 2010ultra GC-MS (Shimadzu, Duisburg, Germany) according to a protocol given elsewhere [44]. The gas chromatograph was equipped with a ZB-5MS fused silica capillary column (30 m × 0.25 mm ID, df = 0.25 μm) from Phenomenex (Aschaffenburg, Germany). The extracted mites were dried at 60 °C until weight constancy and the dry weight was determined with a microbalance (Mettler Toledo, XS3DU, 0.1 μg readability and 1 μg repeatability) to calculate the secretion amount per animal/dry weight in [ng/μg], see [44]. Furthermore, we used the chemical data to quantify the relative amounts in [%] of all oil gland compounds based on peak areas. All compounds have been identified previously (for details see [44]), and were assigned using their retention indices and diagnostic ions.

Additionally, 100 specimens per resource were chemically disarmed using an established hexane-recovery-hexane (HRH) protocol which does not influence the mites’ life-history, but results in the complete depletion of the glands ([38]; see Fig. 1). Mites were redistributed to culture plates (*n* = 10 plates per resource, with 10 individuals per plate resulting in 100 samples) and fed with the same resources mentioned above. After 28 days, specimens were individually extracted in hexane to measure the proportion of specimens that had regenerated oil-gland secretions (see [45]; Table 1).

#### Life history experiments

Individual mites (= mothers, n = 10*25) could lay eggs for ten days and the same food and water was provided ad libitum three times a week (see Fig. 1). Every box was checked daily, and we counted the number of eggs, juvenile instars (i.e. larva, protonymph, deutonymph, tritonymph) and adults for a period of up to 12 weeks. We removed freshly hatched adults from the culture plates to ensure no new egg deposition and froze them for further analysis. The total developmental time [days] for each mother’s offspring was calculated as weighted arithmetic mean (developmental time = ∑ [d_i_*p_i_]; where d_i_ is the experiment day and p_i_ is the proportion of new adult specimens on d_i_). Replicates where all offspring died before reaching adulthood were not used to calculate the developmental time (*n* = 72). In addition to the counted data (see Table 1) we dried all individuals at 60 °C to determine the total biomass output of individual mothers, expressed as dry weight [mg] per female.

#### Morphological analysis

The removed mothers from the life-history experiments were used for morphological analysis (see Fig. 1, Table 1), except for damaged individuals that were excluded from the measurements. We overall measured eleven continuous variables of individual specimen (five dorsal and six ventral distances) for the morphometric analysis (see Additional file 2 for details) using a VHX-5000 microscope (Keyence Deutschland GmbH, Neu-Isenburg, Germany) equipped with the VH-Z50L lens. To remove the effect of isometric body size scaling on morphometrical measures we standardized all values of one individual by its notogaster length (see Additional file 2). Afterwards, all specimens (mothers) were dried at 60 °C and the individual body mass, expressed as dry weight [μg] was determined as described above.

#### Analyses of nutritional quality

For C/N analyses dried resource powders (5 ± 1 mg) were weighed into tin capsules. Total organic carbon and nitrogen contents were measured by an elemental analyzer (EA 1108 Elemental Analyser, Carlo Erba, Milan, Italy). Acetanilide (Merck, Darmstadt, Germany) was used as standard. Carbon and nitrogen amounts were calculated based on the standard and the initial dry weight and expressed as C/N ratios (Table 2).

**Table 2:** Carbon to nitrogen ratios (mean ± standard deviation; *n* = 3 replications) of the ten food resources sorted by C/N-ratio, beginning with the lowest (= highest nitrogen content)

	C/N *mean ± SD*
blood	3.3 ± 0.017
bone	4.2 ± 0.042
spirulina	4.3 ± 0.003
chlorella	5.4 ± 0.023
hemp	5.6 ± 0.048
lupine	7.0 ± 0.033
yeast	7.5 ± 0.027
fungi	11.3 ± 0.005
wheat	12.4 ± 0.042
pollen	13.6 ± 0.252

### Data analysis

We analyzed the univariate traits (see Table 1) using Kruskal-Wallis tests and Levene tests to access the overall differences and the variance among resources, respectively. Scatter plots showing all data points and individual posthoc comparisons (Dunn’s test [46] and false discovery rate [47] to correct for multiple tests as implemented in “PMCMR”) for each trait-resource combination can be found in the Additional files 3 and 4. For multivariate traits [=chemical composition (as Bray-Curtis similarities) and morphometric measures (as Euclidean distances)] we also analyzed the differences and variances among resources using PERMANOVA [48] and PERMDISP [48], respectively as implemented in “vegan”. Both multivariate traits were ordinated using discriminant analysis of principal components (DAPC; see [49]) using the package “adgenet”. DAPC transforms the original data by principal component analysis (PCA) prior to the discriminant analysis. We retained 6 (for chemical composition) and 7 (for morphometry) PC-axes based on their Eigenvalues (> 1) and the explained variance (total cumulative variance > 95%).

To statistically access the optimality response of a certain trait to the resource quality parameter (i.e., C/N-ratio) we fitted a non-linear regression model (t’ ~ C/N + C/N^2^; where t’ is a given trait value), using the lm-function in *R Prior* to this statistical analysis we had to adjust our data in several ways: first we transformed the trait data (see results section) to ensure the normality of residuals of every regression and to break potential mean-variance relationships; secondly, we rescaled all the transformed trait values between 0 and 1 using the minimum-maximum normalization (eq. 1)

$$ (1)\ {t}^{\prime }=\frac{t-{t}_{min}}{t_{max}-{t}_{min}} $$

where *t* is any transformed trait value, *t*_*min*_ and *t*_*max*_ are the lowest or highest expression level of a given trait, respectively and *t’* is the min-max normalized trait value. The rescaling was necessary to plot all the optima curves simultaneously and elucidate potential trade-offs among the trait-resource combinations.

To quantify the trait (Table 1) variation for all ten resource treatments and every trait we calculated ln CV (coefficient of variation) according to Nakagawa et al. [50] using eq. (2)

$$ (2)\ln CV=\ln \sigma -\ln \overline{x}+\frac{1}{2\left(n-1\right)} $$

where $\overline{x}$ is the sample mean, σ is the standard deviation and n is the sample size, yielding nine different trait ln CVs for each resource. To summarize the multivariate traits (chemical composition and morphometry) we first calculated the ln CVs for each single component and subsequently averaged the single ln CVs to obtain the mean ln CV for both traits. We used a Kruskal-Wallis test to check whether the nine trait ln CVs across all resources are prone to more/less variation compared to others and if trait types (chemical, life history, morphology) possess different variability using the mean across resource ln CVs. We further analyzed the mean trait variation across traits to test whether some resources produce more variable traits than others and if the variation of trait variation differs across resources using a Kruskal-Wallis and Levene test, respectively. Afterwards, we used these values to calculate the mean ln CV for each resource (see last row of Table 3), to eventually test for optimality of C/N-ratios using a non-linear regression model (using the package “Rcmdr”). Additionally, we used the same analysis to test the influence of the C/N-ratios on the ln CVs of the nine individual traits. Finally, we accessed the relationship of mean and variance of the different traits across all resources by using the mean normalized trait value *t’*_*mean*_ and the ln CV of the univariate traits (because multivariate traits do not have a mean t’). Because the different traits within one resource treatment are not independent from each other we used a linear mixed effect model (LMM) with trait variation as dependent, trait mean as independent and resource treatment as random variable using the R packages “lme4” and “car”. The model was checked for normality of residuals and variance homogeneity, and no further transformation was necessary.

**Table 3:** Character values of the nine selected traits across the ten resources

	blood	bone	chlorella	fungi	hemp	lupine	pollen	spirulina	wheat	yeast
	mean ± SD (CV)	mean ± SD (CV)	mean ± SD (CV)	mean ± SD (CV)	mean ± SD (CV)	mean ± SD (CV)	mean ± SD (CV)	mean ± SD (CV)	mean ± SD (CV)	mean ± SD (CV)
chemistry										
*amount [ng/μg]*	2 ± 2 (109)	2 ± 1 (46)	4 ± 2 (64)	6 ± 3 (52)	2 ± 1 (45)	4 ± 2 (54)	2 ± 1 (54)	8 ± 10 (133)	3 ± 2 (57)	4 ± 3 (65)
*regeneration [%]*	0 ± 0 (0)	75 ± 15 (21)	46 ± 37 (79)	58 ± 25 (42)	90 ± 13 (15)	77 ± 17 (23)	74 ± 28 (38)	91 ± 10 (11)	86 ± 15 (17)	85 ± 15 (17)
*compositon*										
HMBD [%]	4 ± 3 (67)	3 ± 1 (45)	7 ± 12 (166)	12 ± 30 (251)	4 ± 2 (46)	5 ± 8 (170)	6 ± 2 (26)	5 ± 5 (96)	6 ± 2 (36)	6 ± 1 (20)
NER [%]	13 ± 8 (63)	8 ± 7 (87)	19 ± 9 (48)	20 ± 21 (104)	11 ± 3 (29)	12 ± 16 (128)	11 ± 4 (36)	11 ± 8 (70)	16 ± 9 (56)	17 ± 4 (25)
NYF [%]	36 ± 9 (25)	39 ± 11 (27)	29 ± 5 (18)	34 ± 6 (17)	43 ± 5 (12)	41 ± 10 (25)	35 ± 5 (14)	37 ± 10 (26)	35 ± 12 (35)	30 ± 5 (16)
C13 [%]	5 ± 2 (29)	12 ± 4 (32)	9 ± 5 (54)	9 ± 8 (85)	8 ± 5 (56)	10 ± 5 (50)	15 ± 5 (34)	10 ± 6 (62)	7 ± 2 (32)	7 ± 2 (23)
ACA [%]	16 ± 4 (27)	14 ± 5 (36)	21 ± 5 (26)	21 ± 6 (29)	16 ± 5 (31)	15 ± 6 (40)	13 ± 5 (35)	18 ± 6 (33)	18 ± 6 (31)	23 ± 4 (17)
C15 [%]	12 ± 3 (29)	13 ± 3 (27)	12 ± 4 (37)	12 ± 5 (41)	9 ± 9 (104)	12 ± 4 (33)	9 ± 3 (30)	12 ± 5 (43)	11 ± 2 (21)	10 ± 2 (25)
C17 [%]	14 ± 5 (35)	12 ± 3 (22)	8 ± 5 (69)	5 ± 6 (127)	9 ± 2 (23)	9 ± 4 (38)	11 ± 3 (27)	7 ± 4 (66)	8 ± 2 (24)	6 ± 1 (23)
life-history										
*dev. time [days]*	37 ± − (−)	68 ± 11 (15)	56 ± 8 (15)	71 ± 7 (9)	49 ± 7 (15)	63 ± 5 (8)	58 ± 8 (14)	75 ± 6 (9)	59 ± 7 (11)	48 ± 3 (7)
*survival [%]*	8 ± 28 (30)	37 ± 31 (49)	8 ± 13 (14)	15 ± 16 (18)	50 ± 29 (58)	67 ± 29 (89)	51 ± 26 (54)	11 ± 16 (18)	78 ± 18 (79)	49 ± 18 (36)
*offspring [N]*	0.1 ± 0.3 (332)	8 ± 10 (127)	2 ± 3 (190)	2 ± 2 (102)	14 ± 14 (101)	19 ± 12 (62)	11 ± 9 (81)	3 ± 5 (154)	45 ± 26 (59)	22 ± 10 (45)
*output [mg]*	0.001 ± 0.002 (332)	0.7 ± 0.8 (119)	0.3 ± 0.3 (125)	0.2 ± 0.1 (77)	1.1 ± 0.9 (85)	1.5 ± 0.5 (38)	0.7 ± 0.6 (83)	0.3 ± 0.5 (171)	2.2 ± 1.1 (46)	1.3 ± 0.6 (44)
morphology										
*body mass [μg]*	8 ± 4 (46)	17 ± 6 (33)	27 ± 9 (34)	15 ± 4 (30)	19 ± 4 (23)	17 ± 3 (21)	14 ± 6 (42)	19 ± 5 (25)	14 ± 3 (24)	25 ± 8 (35)
*morphometry*										
Nw I [% NL]	92 ± 16 (17)	88 ± 6 (7)	92 ± 10 (11)	102 ± 15 (15)	87 ± 15 (17)	83 ± 19 (23)	87 ± 9 (10)	93 ± 16 (17)	91 ± 10 (11)	92 ± 14 (15)
Nw II [% NL]	63 ± 10 (16)	58 ± 5 (9)	62 ± 8 (13)	61 ± 8 (14)	59 ± 6 (11)	56 ± 13 (23)	58 ± 5 (9)	53 ± 8 (15)	60 ± 6 (11)	60 ± 10 (17)
PL [% NL]	32 ± 11 (36)	25 ± 8 (32)	27 ± 8 (32)	35 ± 11 (32)	25 ± 8 (31)	23 ± 10 (44)	26 ± 9 (33)	30 ± 12 (41)	28 ± 9 (31)	27 ± 10 (37)
Bd [% NL]	35 ± 8 (24)	33 ± 5 (15)	31 ± 5 (15)	31 ± 7 (21)	32 ± 8 (25)	30 ± 8 (28)	33 ± 5 (15)	31 ± 8 (26)	33 ± 7 (23)	28 ± 6 (21)
GL [% NL]	27 ± 4 (15)	26 ± 3 (10)	26 ± 3 (12)	28 ± 5 (17)	25 ± 5 (18)	24 ± 6 (25)	26 ± 3 (11)	27 ± 4 (16)	27 ± 4 (14)	27 ± 4 (16)
Gw I [% NL]	20 ± 4 (21)	20 ± 3 (16)	17 ± 3 (16)	22 ± 6 (28)	16 ± 3 (17)	18 ± 5 (27)	20 ± 3 (16)	21 ± 5 (24)	20 ± 4 (19)	19 ± 4 (21)
Gw II [% NL]	14 ± 3 (20)	13 ± 2 (17)	11 ± 2 (20)	13 ± 4 (31)	11 ± 2 (20)	11 ± 3 (30)	11 ± 2 (21)	13 ± 3 (23)	12 ± 1 (13)	12 ± 3 (24)
AL [% NL]	38 ± 7 (19)	36 ± 4 (12)	39 ± 7 (17)	44 ± 8 (18)	40 ± 8 (19)	37 ± 9 (24)	37 ± 6 (17)	40 ± 6 (16)	39 ± 6 (16)	42 ± 9 (21)
Aw I [% NL]	9 ± 2 (23)	9 ± 2 (24)	8 ± 2 (21)	9 ± 2 (24)	10 ± 3 (28)	8 ± 2 (26)	8 ± 2 (23)	8 ± 2 (20)	8 ± 1 (14)	9 ± 2 (18)
Aw II [% NL]	14 ± 3 (23)	14 ± 3 (21)	13 ± 3 (19)	15 ± 4 (26)	15 ± 3 (18)	12 ± 4 (28)	12 ± 2 (19)	14 ± 3 (23)	12 ± 2 (16)	15 ± 4 (24)
mean ln CV^a^	−0.23	−0.93	−0.78	−0.97	−1.03	−1.05	−0.92	−0.92	−1.13	−1.27

Traits in *italics* denote the categories used for the trait variation analyses. Numbers represent means with standard derivations (SD) and raw coefficients of variation (CV). Abbreviations: HMBD = 2-hydroxy-6-methylbenzaldehyde, NER = neral, NYF = neryl formate, C13 = tridecane, ACA = γ-acaridial (3-hydroxybenzene-1,2-dicarbaldehyde), C15 = 7-pentadecene/pentadecane, C17 = 6,9-heptadecadiene/8-heptadecene/heptadecane; NL = notogaster length, Nw I = notogaster width I, Nw II = notogaster width II, PL = prodorsum length, Bd = bothridial setae distance, GL = genital plate length, Gw I = genital plate width I, Gw II = genital plate width II, AL = anal plate length, Aw I = anal plate width I, Aw II = anal plate width II. - = not applicable. ^a^ The mean ln CV per resource was calculated based on eq. (2) using the individual ln CVs of all traits from one resource

The following replicates were not included in the statistical analyses: chemical samples with contaminations for secretion amounts (*n* = 14 of 300); mothers which did not survive the egg laying period for life history data (*n* = 34 of 250); total biomass output lower than 1 μg (not reliably measurable, *n* = 8 of 250); non intact mothers from mass measurement (n = 8 of 250); damaged specimen for morphometric measures (*n* = 47 of 250). The raw data is deposited in Additional file 4. All statistical analyses were performed with R 3.3.2 [51], using the packages “adegenet” [49], “car” [52], “lme4” [53], “PMCMR” [54], “vegan” [55], and “Rcmdr” [56].

## Results

### Nutritional quality

Some food resources were characterized by an extreme composition – e.g. blood meal had a very high C/N ratio, which corresponds to a high nitrogen content (13.3 ± 0.1% N; mean ± SD), while pollen was nitrogen poor, but comparatively carbon rich (3.6 ± 0.1% N; mean ± SD). Yet, most of the remaining resources had a quite equilibrated stoichiometry (Table 2). Thus, the variability of the C/N ratios across the food was high (CV = 47%; 7.5 ± 3.5; mean ± SD), and the spanned from 3.3 (blood meal) to 13.6 (pollen). While we focused our analyses on C/N ratios only, the foods also differed in other nutritional dimensions (i.e. elements and macronutrients; see Additional file 1), but their influence is beyond this study.

### Chemical traits

**Fig. 2:**
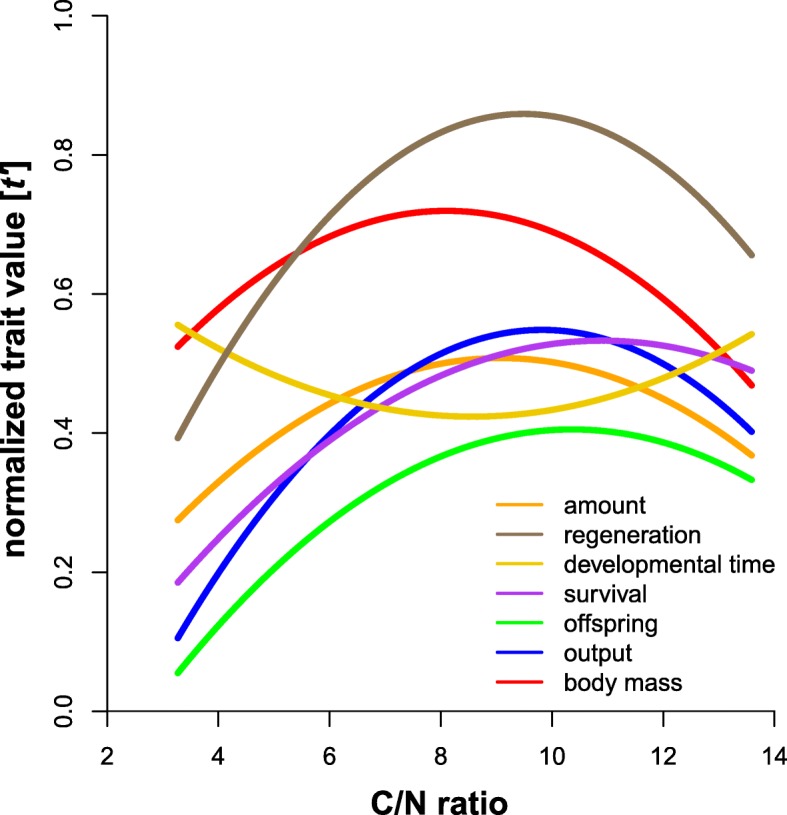
The effect of the elemental composition of the food (C/N-ratios) on the mean expression value of multiple univariate traits of the ‘phenotypically tractable’ model mite lineage *Archegozetes longisetosus* ran. Curves are regressions of non-linear models (Table 4). The y-axis denotes the normalized trait value (between 0 and 1) for each trait after normality transformation (see Table 4) and rescaling (min-max normalization; see eq. 1). Amount = secretion amount [ng/μg] of individual mites; regeneration = fraction of regenerating individuals [%]; developmental time = time [days] of one females offspring from egg to adult, calculated as weighted mean; survival = survival rate of one females offspring [%]; offspring = total number of offspring per female [N*female^− 1^]; output = the reproductive biomass output per female [mg*female^− 1^] and body mass = body mass [μg] of individual mites raised on the ten resources. Scatter plots for each trait across the resources as well as detailed post-hoc comparisons using Dunn’s test can be found the Additional files 3 and 4, respectively

**Fig. 3:**
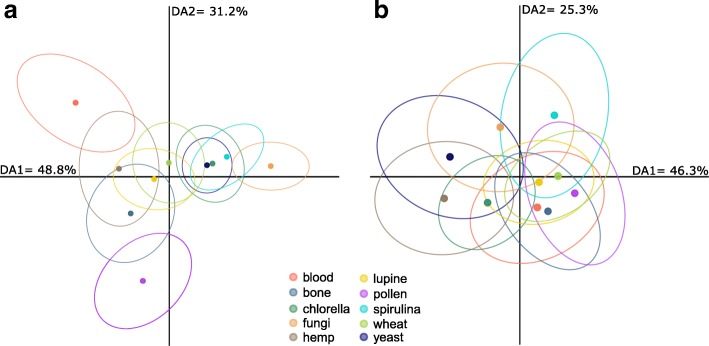
Ordination (discriminant analysis of principal components) of the relative composition of defensive gland exudates [%] (**a**), as well as the eleven morphometric characters [% relative to notogaster length] (**b**) of mite individuals reared on the ten resources. The filled circles mark the group mean (centroid) of each respective group, ellipsoid hulls indicate the group dispersion (multivariate variance) as 95% confidential space. Percentages denote the variance explained by each axis. Colors correspond to the figure legend and Fig. 1

Three traits were related to chemical defense (Table 1). The individual amount of defensive secretions (ng/μg; Kruskal-Wallis: *n* = 286, df = 9, χ^2^ = 62.74, *p* <  0.0001; Table 3; Additional file 3) and its variation within each group (Levene: F_9,276_ = 11.25, *p* <  0.0001; Table 3) differed across all resources. The fraction of regenerating individuals (%; Kruskal-Wallis: *N* = 100, df = 9, χ ^2^ = 42.97, *P* <  0.0001; Table 3; Additional file 3) and their variation within each group (Levene: F_9,90_ = 2.99, *P* = 0.004; Table 3) differed across all resources. Both univariate chemical traits, the individual secretion amount (F_2,283_ = 16.84, *r*^*2*^ = 0.11) and fraction of regenerating individuals (F_2,97_ = 11.01, *r*^*2*^ = 0.19) showed C/N optima curves (Fig. 2; for detailed statistics of linear and nonlinear effect estimates see Table 4). Also, the relative composition (%; Fig. 3a; Table 3) of the seven compounds found in the defensive secretions showed differences among groups (PERMANOVA: pseudo-F_9,276_ = 14.01, *r*^*2*^ = 0.31, *p* <  0.0001) as well as in multivariate dispersion (=variation; PERMDISP: F_9,276_ = 3.91, *p* <  0.001; see ellipsoid sizes in Fig. 3a) and showed a significant nonlinear response to food C/N-ratios (Table 4).

**Table 4:** The linear and non-linear effects of food C/N ratios on the nine different traits of the oribatid mite *Archegozetes longisetosus*

	Linear effect	Nonlinear effect
	C/N	C/N x C/N
Chemistry		
Amount (log transformation)		
Estimate^a^ ± SE	0.125 ± 0.023	−0.007 ± 0.001
t_286_	5.546	−5.242
p	< 0.0001	< 0.0001
Composition (1/4-power transformation)		
*pseudo*F_286_^b^	354.390	118.350
*r*^2^	0.469	0.157
p	< 0.0001	< 0.0001
Regeneration (arcsine square root transformation)		
Estimate^a^ ± SE	0.229 ± 0.057	−0.012 ± 0.003
t_100_	4.031	−3.641
p	0.0001	0.0004
Life history		
Developmental time (no transformation)		
Estimate^a^ ± SE	−0.081 ± 0.035	0.005 ± 0.002
t_172_	−2.321	2.455
p	0.0215	0.0151
Survival (arcsine square root transformation)		
Estimate^a^ ± SE	0.130 ± 0.039	− 0.006 ± 0.002
t_216_	3.357	−2.693
p	0.0009	0.0077
Offspring (square root transformation)		
Estimate^a^ ± SE	0.144 ± 0.029	−0.007 ± 0.002
t_216_	4.906	−4.140
p	< 0.0001	< 0.0001
Output (square root transformation)		
Estimate^a^ ± SE	0.203 ± 0.033	− 0.010 ± 0.002
t_208_	6.191	−5.530
p	< 0.0001	< 0.0001
Morphology		
Body mass (log transformation)		
Estimate^a^ ± SE	0.135 ± 0.021	−0.008 ± 0.001
t_208_	6.543	−7.082
p	< 0.0001	< 0.0001
Morphometry (1/4-power transformation)		
*pseudo*F_201_^b^	0.960	2.781
*r*^2^	0.005	0.013
p	0.3457	0.0603

^a^The estimate of the linear effect describes the slope of the relationship between the C/N ratio and the response variable (i.e. normalized trait value), while the estimate of the non-linear effect is the curvature of the quadratic relationship (i.e. normalized trait value ~ C/N x C/N). ^b^ For the multivariate traits we used permutational multivariate analysis of variance (PERMANOVA), instead of a normal linear model

### Life-history traits

In total, we investigated four life-history traits, related to reproductive fitness and resource allocation (Table 1). The developmental time of each females’ offspring, calculated as weighted mean (days; Kruskal-Wallis: *n* = 172, df = 8, χ ^2^ = 100.54, *p* <  0.0001; Table 3; Additional file 3) and its variation within each group (Levene: F_8,163_ = 3.22, *p* = 0.002; Table 3) differed across all resources. Blood meal was excluded from the analysis, because only one individual developed from egg to adult. While stock cultures with blood meal are stable, individual rearing seemed problematic. The survival of each females’ offspring (%; *n* = 216, df = 9, χ ^2^ = 112.47, *p* <  0.0001; Table 3; Additional file 3) and its variation within each group (Levene: F_9,206_ = 4.02, p <  0.0001; Table 3) differed across all resources. The total number of offspring per female (N*female^− 1^; Kruskal-Wallis: n = 216, df = 9, χ 2 = 134.80, p <  0.0001; Table 3; Additional file 3) and its variation within each group (Levene: F_9,206_ = 7.97, p <  0.0001; Table 3) differed significantly across all resources. The reproductive output per female (mg*female^− 1^; Kruskal-Wallis: *n* = 208, df = 9, χ ^2^ = 125.19, p <  0.0001; Table 3; Additional file 3) and its variation within each group (Levene: F_9,198_ = 5.04, *p* <  0.0001; Table 3) also differed across all resources. All life-history traits responded with optima curves (Fig. 2) to the C/N-ratio gradient (Table 4; developmental time: F_2,169_ = 3.44, *r*^*2*^ = 0.04; survival of one females’ offspring: F_2,213_ = 16.27, *r*^*2*^ = 0.13; total number of offspring per female: F_2,213_ = 25.79, *r*^*2*^ = 0.20; reproductive output per female: F_2,205_ = 28.74, *r*^*2*^ = 0.22).

### Morphological traits

**Fig. 4:**
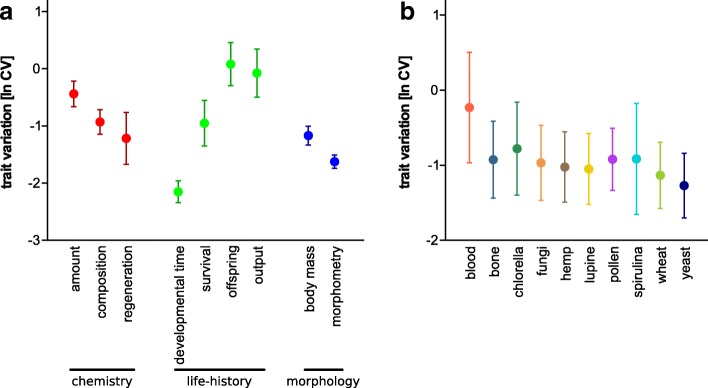
Trait variation [ln CV] of the nine selected traits (Table 1) across all ten resources (**a**) and the overall trait variation [ln CV] of each resource across all traits (**b**). For (**a**), individual traits are grouped into their respective category (chemistry, life-history or morphology). Circles represent means, lines denote the standard error, colors in (**b**) correspond to Fig. 1

Body mass and various morphometric measurements describing body shape were included (Table 1, Fig. 4). The individual body masses (μg; Kruskal-Wallis: n = 208, df = 9, χ ^2^ = 89.06, p <  0.0001; Table 3) and their variation within each group (Levene: F_9,198_ = 2.60, *p* = 0.007; Table 3) differed across all resources and again followed a C/N optimum (Fig. 2; Table 4; F_2,205_ = 30.92, *r*^*2*^ = 0.23). The eleven morphometric characters (% NL; Table 3; see Additional file 1 for character overview) measured for individual mites showed moderate, yet differences among groups (PERMANOVA: pseudo-F_9,192_ = 2.36, *r*^*2*^ = 0.10, *p* = 0.003; Fig. 3b), but no differences in multivariate dispersion (=variation; F_9,192_ = 1.51, *p* = 0.147; compare ellipse sizes in Fig. 2b) and no nonlinear response to food C/N-ratios (Table 4).

### Trait variation

**Fig. 5:**
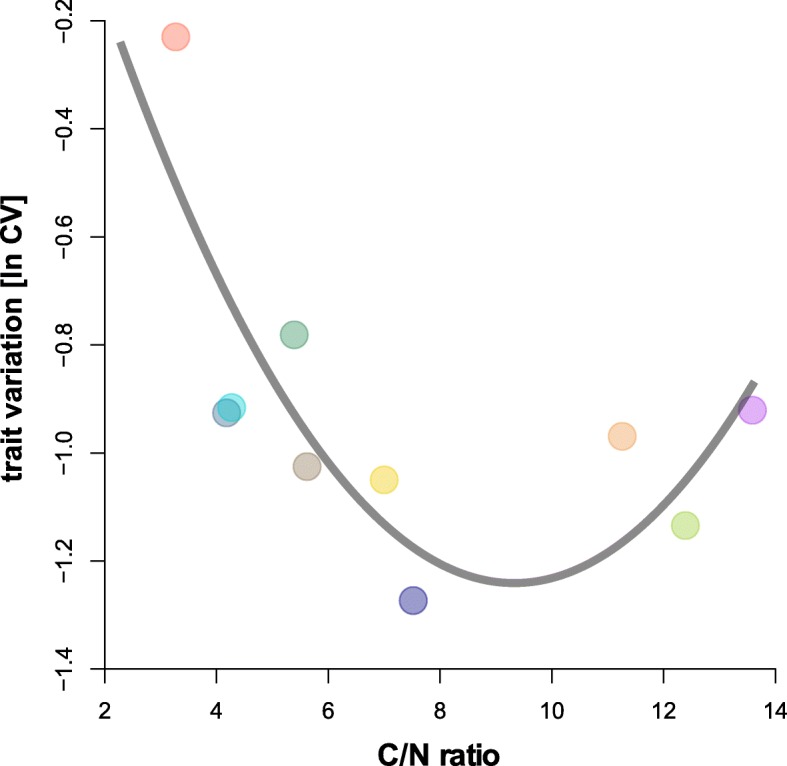
The relationship of the mean trait variation [ln CV] of one resource across all traits plotted against the C/N ratio. The grey curve denotes a quadratic regression (linear effect – C/N, estimate: − 0.38 ± 0.12, *t* = − 3.2, *p* = 0.015; nonlinear effect – C/N x C/N, estimate: 0.02 ± 0.01, *t* = 2.9, *p* = 0.022). Circles represent means, colors correspond to Fig. 1

**Fig. 6:**
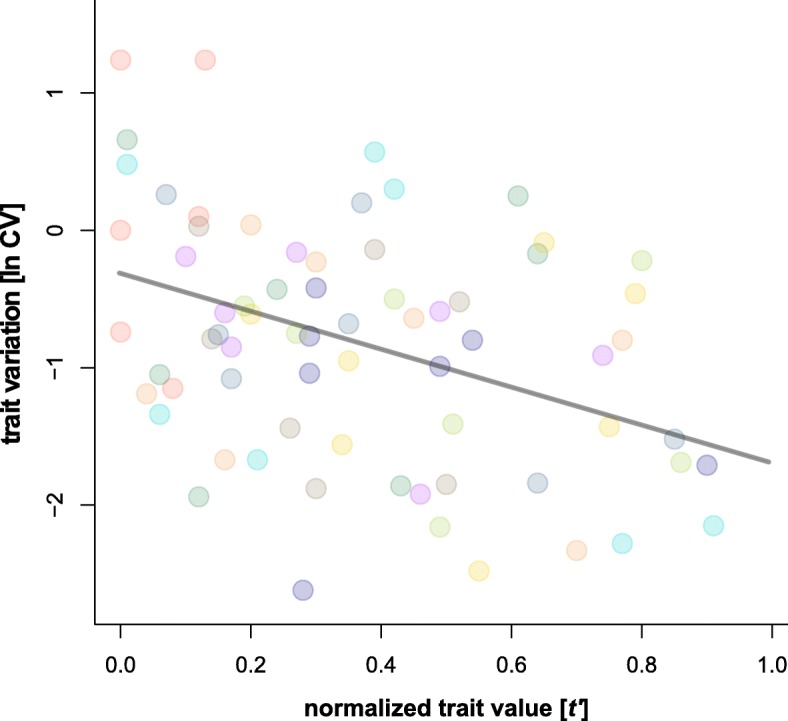
The relationship of trait means [normalized trait value t’] to trait variation [ln CV] of the different univariate traits across resources. The grey line denotes the linear regression based on a mixed linear model (intercept, estimate: − 0.36 ± 0.17, *t* = − 2.1, *p* = 0.040; slope, estimate: − 1.27 ± 0.38, *t* = 3.3, *p* = 0.002). Colors of the circles correspond to Fig. 1

All traits (Table 1) were influenced by diet (Figs. 2 and 3; Table 3) and varied across all resources (for untransformed CVs [%] see Table 3). Also, ln CVs of the traits (Table 1) differed (Kruskal-Wallis: *n* = 89, df = 8, χ ^2^ = 59.92, p <  0.0001; Fig. 4a). Generally, the variability was the same among the trait types (Kruskal-Wallis: *n* = 9, df = 2, χ ^2^ = 1.75, *p* = 0.41). Life-history traits had a ln CV_mean_ = − 0.78 (ln CVs for dev. time, survival, offspring and output were − 2.15, − 0.95, 0.08 and − 0.08, respectively), chemical traits responded with a ln CV_mean_ = − 0.86 (ln CVs for amount, composition and regeneration were − 0.44, − 1.22 and − 0.93, respectively) and the morphological traits showed a ln CV_mean_ = − 1.40 (ln CVs for body mass and morphometry were − 1.17 and − 1.63, respectively) across all ten feeding treatments. There were no differences among mean trait variations across resources (Kruskal-Wallis: n = 89, df = 9, χ ^2^ = 5.28, *p* = 0.81; Fig. 4b), also the variance of trait variation was not heteroscedastic among resources (Levene: F_9,79_ = 1.64, *p* = 0.21). We also tested whether the mean total variation of one resource (across all traits) is related to the C/N ratio of the food (Table 2) and found that trait variation responded to the C/N-ratio of the food as an optimum curve (*r*^2^ = 0.54, F_2,7_ = 6.36, *p* = 0.027: Fig. 5). For the individual traits’ ln CVs, however, only the number of offspring and the biomass output per female responded optimally (*r*^2^ = 0.71, F_2,7_ = 8.63, *p* = 0.013 and *r*^2^ = 0.83, F_2,7_ = 16.45, *p* = 0.002, respectively), while the traits showed no optimum related to C/N-ratios (all *p* > 0.15). Eventually, there was a negative relationship of mean and the variance of the different univariate traits (Fig. 6; LMM: F_1,59_ = 10.89, p = 0.002).

## Discussion

Dietary induced intraspecific reaction norms and variation are a mostly neglected topic in evolutionary ecology [2, 34]. Over 100 years ago, Woltereck [34] started to discuss underlying mechanisms, but also noted that it is hardly possible to quantify the range of a species’ variability caused by nutritional quality, because even in pure linages of facultative parthenogenetic species, like *Daphnia*, a full control of the genotype is not possible. He also suggested that an obligate asexual species would be a perfect model to study nutritional reaction norms of traits and their plasticity. That is why parthenogenetic generalists (like some oribatid mites), which can tolerate a wide range of environmental conditions (i.e. possess a general purpose genotype; [57]), are the models that Woltereck [34] proposed. Correspondingly, we used the single genotype of a “phenotypically-tractable” model lineage *A. longisetosus* ran to unravel the relationship of food quality and variation of multiple traits (i.e. phenotypic plasticity).

### Nutritional effects on traits and variation

Most studies on a single species so far focused on the relationship of nutrients to one or a distinct set of traits – often times related to fitness (e.g., [18, 20, 58, 59]). We think that our multiple traits approach – also including presumable neutral characters – better allows to disentangle patterns and mechanisms of nutrient influence on the mean and variance as well as the inherent plasticity of traits. In our experiment, all traits responded to diet and showed quadratic reaction norms (= optima) of different strength (Fig. 2). Physiological theory formally conceptualized this quadratic (or concave) response of fitness relevant performance traits as “threshold elemental ratio” [for element stoichiometry; 26] or as “Bertrand’s rule” for the concentration of essential micronutrients [60] and also macronutrients [18]. While the first concept is a stoichiometric approach based on the proportional relationship of elements, the latter uses absolute concentrations or the amount of nutrient intake to explain reaction norms of individuals [18, 20, 26, 31]. Yet, both concepts are unified by a similar prediction: if a food is too unbalanced (containing too low levels of a certain nutrient or element) to reach the intake target, the benefits gained from increasing the amount or ratio of this nutrient or element also increases until they reach an equilibrium. More nutrients/elements beyond this threshold (the intake target) are associated with increasing costs for the regulatory mechanisms resulting in physiological disadvantage higher than the original benefit [20, 31]. Our results for C/N show that a simple threshold elemental ratio (presumably close to our model species intake target) applies to multiple traits and, even more important, also to their variability and thus to phenotypic plasticity. This is because the variability of each trait across resources was heteroscedastic, indicating that food quality not only changed the mean (e.g., [61, 62]), but also the variation of a trait [25, 28–30]. These findings could help to propose a general eco-physiological mechanism causing dietary related intraspecific trait variation derived for our model system: the low performance and high plasticity of animals feeding on resources from the “edges” of an elemental or nutritional gradient ingest imbalanced food with stoichiometric shortcomings which causes stress related costs to deal with the deficits as well as surpluses of elements or nutrients [18, 26]. While at low N-content (high C/N ratio) trait performance was limited by a short supply of protein, mites shifted to a C-limited trait performance at high level of N (low C/N). Consequently, high variability of all traits (i.e. high phenotypic plasticity) occurred if either protein (N) or energy (C) limited the formation and performance of traits. More general (see Fig. 6): if there is a considerable trade-off between consumed surplus and deficit nutrients in one resource, the variation across multiple traits within a phenotype is high, while the trait mean is low. Reciprocally, trait variation is low, and the trait mean is high at nutritional optima where no essential nutrient is in short supply and a respective genotype can almost bear its full potential. This connection of trait means and variances across resources (expressed as a direct negative relationship, Fig. 6) is further evidence for the wide applicability of the balanced diet hypothesis [25]. The concave responses of all traits and their variability to overall food quality (C/N ratio) in our experiments not only indicated that the threshold-elemental-ratio-rule applied for a wide range of traits and their variability, but further suggest costs [20] to maintain a high mean and low variance of a trait. This means that mites feeding on “edge” resources must deal with high costs during allocating resources compared to mite consuming the “optimum” food. Despite these costs, however, variation at the “edges” may still be beneficial, because it enables at least a small number of individuals to survive unfavored conditions. For instance, a recent synthesis by Forsman and Wennersten [63] found that variation seems to be more important under stressful circumstances when animals are forced to exist under suboptimal conditions - like stoichiometrically imbalanced food not meeting the intake target [26] - and may enable the survival of a population [64].

### Inherent variability of traits

Generally, there are no multicellular organisms without a certain plasticity, because intra-individual trade-offs as reactions towards environmental conditions like temperature, salinity or resource availability, but also biotic factors like predation will lead to variation of traits [15, 63, 65]. Besides selection, it is still poorly understood why some traits are more prone to variation than others, i.e. bear higher plasticity. Our data indicates that some traits tend to be more variable than other, but this was not related to certain “trait types” (in our case “chemistry”, “life history” and “morphology”). This different *variability potential* allows to derive different hypothesis: a certain trait may react with higher variability to an environmental gradient, because it faces more trade-offs along this gradient than other characters; or the formation/development of a trait may be more “complex” and thus demands a stronger segregation of energy leading to more trade-offs. Consequently, lower trait variation may indicate less trade-offs (or selection) along a certain gradient. Also, a lower variability may be a signal for an inherently lower plasticity of a trait because it is less controlled by the phenotypes’ response or selection. For instance, in our experiments, the body size of the mites changed considerably along the C/N gradient, yet the overall body shape (morphometric measurements) only showed a weak response and low variability. This may indicate, that – besides selection or genetical conservation – the overall body size could be influenced by the phenotypic response to altered nutrients. Yet, the proportions of the body shape isometrically scaled with this phenotypic change, leading to low overall variability.

## Conclusions

Overall, we have shown that the threshold elemental ratio rule [18, 26, 60], applies to a wide range of traits and also to dietary caused intraspecific variation of multiple traits (= phenotypic plasticity) in a model system excluding genotypic variation. Thus, there seems not only to be a nutritional optimum for a traits’ mean, but also its variation, which further strengthens the balanced diet hypothesis [25]. Highly imbalanced food results in lower trait means and higher trait variability (Figs. 2, 5, 6), potentially caused by more/stronger trade-offs across resources arising from physiological stress. Additionally, some traits seem to have higher inherent variation than others, irrespective of “trait type”. Based on our empirical evidence we propose the following hypotheses, leading to further experimental evaluation; i) the mean trait variation calculated based on all trait variation within a resource could be used as an indicator for the food quality and ii) given that there is a direct link of both mean and variation of traits (Fig. 6), the latter might also be indicative for trade-offs an animal faces when feeding on imbalanced diets [20]. Finally, using a single parthenogenetic lineage excludes genotypic plasticity and creates a phenotypically tractable model species. This is a first step to establish an experimental platform to ultimately disentangle how variation (or the capacity to express plasticity) affects the fitness of individuals [65].

## Additional files

Additional file 1: Raw data table. (XLSX 560 kb)

Additional file 2: Morphometric data. (PDF 370 kb)

Additional file 3: Trait data used for Fig. 2. (PDF 253 kb)

Additional file 4: Post-hoc test results. (PDF 94 kb)
