# Supplementary material for: Nutritional quality modulates trait variability
Source: Front Zool. 2018 Dec 5;15:50. doi: 10.1186/s12983-018-0297-2 (PMC6282258; doi:10.1186/s12983-018-0297-2)
Supplement: Supplementary file 2 — Morphometric data. (PDF 370 kb) [file 12983_2018_297_MOESM2_ESM.pdf]

Measured distances for morphometric analysis

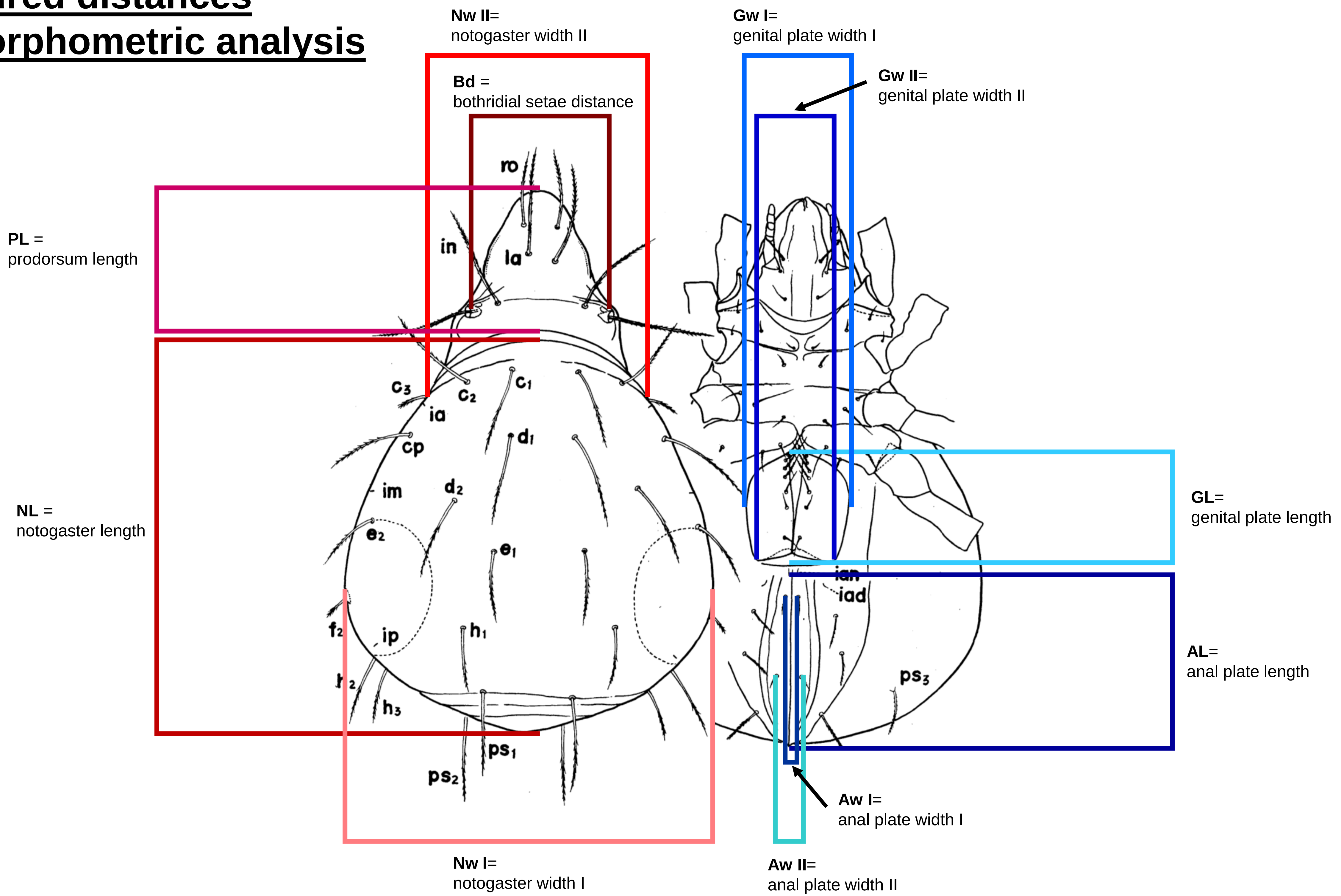

Modified after:
Aoki J. (1965). Oribatiden (Acarina) Thailands I. Nature and Life in Southeast Asia, 4: 129-193.
