# Supplementary figures and images for: Nutritional quality modulates trait variability
Source: Front Zool. 2018 Dec 5;15:50. doi: 10.1186/s12983-018-0297-2 (PMC6282258; doi:10.1186/s12983-018-0297-2)

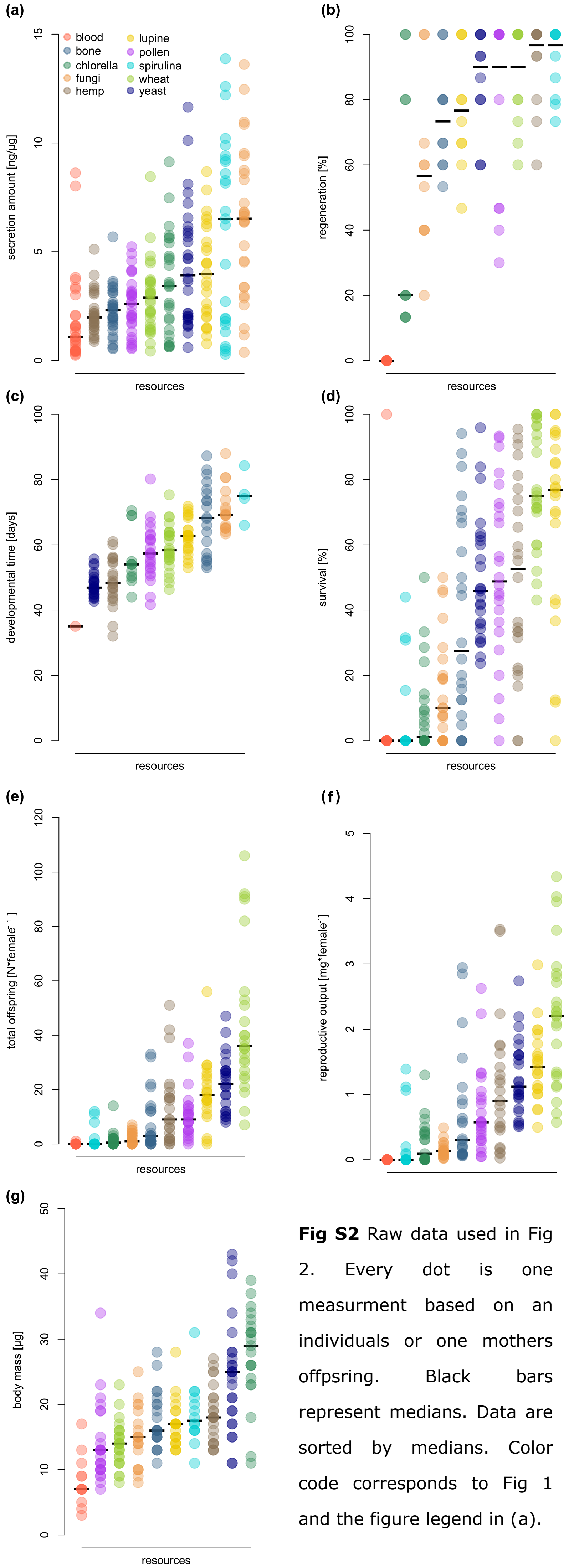

Supplement: Supplementary file 3 — Trait data used for Fig. 2. (PDF 253 kb) [file 12983_2018_297_MOESM3_ESM.pdf]
