# Supplementary material for: Nutritional quality modulates trait variability
Source: Front Zool. 2018 Dec 5;15:50. doi: 10.1186/s12983-018-0297-2 (PMC6282258; doi:10.1186/s12983-018-0297-2)
Supplement: Supplementary file 4 — Post-hoc test results. (PDF 94 kb) [file 12983_2018_297_MOESM4_ESM.pdf]

Secretion amount [ng/µg] Dunn’s posthoc test (false discovery rate corrected):

	blood	chlor	hemp	yeast	bone	lupine	fungi	pollen	spiru
chlor	0.00417	-	-	-	-	-	-	-	-
hemp	0.26029	0.08162	-	-	-	-	-	-	-
yeast	0.00011	0.42059	0.00796	-	-	-	-	-	-
bone	0.15316	0.15934	0.70570	0.01952	-	-	-	-	-
lupine	0.00017	0.44397	0.00985	0.97461	0.02497	-	-	-	-
fungi	2.0e-08	0.01439	1.3e-05	0.09656	4.8e-05	0.09685	-	-	-
pollen	0.06711	0.29261	0.46562	0.05871	0.69711	0.06711	0.00021	-	-
spiru	4.8e-05	0.25366	0.00355	0.69711	0.00985	0.69711	0.21962	0.02530	-
wheat	0.01526	0.62566	0.19797	0.17894	0.34467	0.19797	0.00250	0.59318	0.09656

Regeneration [%]Dunn’s posthoc test (false discovery rate corrected):

	blood	chlor	hemp	yeast	bone	lupine	fungi	pollen	spiru
chlor	0.05669	-	-	-	-	-	-	-	-
hemp	1.6e-05	0.04771	-	-	-	-	-	-	-
yeast	9.8e-05	0.11440	0.76493	-	-	-	-	-	-
bone	0.00286	0.44371	0.31468	0.50775	-	-	-	-	-
lupine	0.00097	0.31468	0.44371	0.70767	0.83992	-	-	-	-
fungi	0.04771	0.92359	0.05634	0.14354	0.49991	0.37591	-	-	-
pollen	0.00058	0.27677	0.50665	0.77142	0.76493	0.90612	0.31468	-	-
spiru	1.6e-05	0.04609	0.92800	0.70767	0.29719	0.41863	0.04771	0.47283	-
wheat	4.8e-05	0.07756	0.83992	0.89471	0.44371	0.57254	0.09871	0.69443	0.80461

Body mass [µg] Dunn’s posthoc test (false discovery rate corrected):

	blood	chlor	hemp	yeast	bone	lupine	fungi	pollen	spiru
chlor	2.9e-10	-	-	-	-	-	-	-	-
hemp	2.9e-05	0.01150	-	-	-	-	-	-	-
yeast	1.2e-08	0.37429	0.07900	-	-	-	-	-	-
bone	0.00046	0.00096	0.47747	0.01385	-	-	-	-	-
lupine	0.00046	0.00096	0.47747	0.01385	0.98999	-	-	-	-
fungi	0.01385	1.9e-05	0.07093	0.00046	0.30210	0.30210	-	-	-
pollen	0.07093	3.1e-08	0.00486	1.9e-06	0.03977	0.03977	0.40469	-	-
spiru	0.00014	0.03004	0.95885	0.15396	0.47747	0.47747	0.09574	0.01245	-
wheat	0.03977	1.4e-07	0.01150	8.0e-06	0.07093	0.07093	0.52979	0.79407	0.02216

Developmental time [days] Dunn's posthoc test (false discovery rate corrected):

	chlor	hemp	yeast	bone	lupine	fungi	pollen	spiru
hemp	0.09575	-	-	-	-	-	-	-
yeast	0.02883	0.55384	-	-	-	-	-	-
bone	0.00848	2.2e-07	2.7e-09	-	-	-	-	-
lupine	0.04661	9.9e-06	1.6e-07	0.37011	-	-	-	-
fungi	0.00041	1.5e-09	1.1e-11	0.29632	0.04661	-	-	-
pollen	0.55675	0.00839	0.00069	0.01212	0.08998	0.00041	-	-
spiru	0.00870	8.6e-05	1.7e-05	0.31838	0.13022	0.70515	0.01505	-
wheat	0.31251	0.00094	5.1e-05	0.04450	0.25582	0.00212	0.55675	0.03282

Survival [%] Dunn's posthoc test (false discovery rate corrected):

	blood	chlor	hemp	yeast	bone	lupine	fungi	pollen	spiru
chlor	0.77731	-	-	-	-	-	-	-	-
hemp	0.00018	3.5e-05	-	-	-	-	-	-	-
yeast	0.00016	2.9e-05	0.98373	-	-	-	-	-	-
bone	0.00670	0.00383	0.23212	0.22463	-	-	-	-	-
lupine	9.8e-07	2.7e-08	0.15476	0.15476	0.00669	-	-	-	-
fungi	0.35988	0.48650	0.00060	0.00051	0.03165	1.1e-06	-	-	-
pollen	0.00013	2.0e-05	0.92596	0.92596	0.18691	0.19758	0.00035	-	-
spiru	0.77731	0.95626	0.00122	0.00111	0.02752	1.5e-05	0.65809	0.00083	-
wheast	5.6e-09	2.1e-11	0.01063	0.01063	0.00015	0.35988	2.1e-09	0.01671	1.6e-07

Total offspring [N*female^{-1}] Dunn's posthoc test (false discovery rate corrected):

	blood	chlor	hemp	yeast	bone	lupine	fungi	pollen	spiru
chlor	0.30727	-	-	-	-	-	-	-	-
hemp	9.1e-05	0.00033	-	-	-	-	-	-	-
yeast	7.2e-09	6.6e-09	0.02925	-	-	-	-	-	-
Bone	0.00570	0.03196	0.16583	0.00027	-	-	-	-	-
lupine	5.3e-07	1.2e-06	0.18735	0.42985	0.00709	-	-	-	-
fungi	0.14257	0.57375	0.00270	1.1e-07	0.12768	1.6e-05	-	-	-
pollen	0.00012	0.00050	0.90848	0.02237	0.19530	0.15954	0.00384	-	-
spiru	0.30727	0.86763	0.00847	1.1e-05	0.13936	0.00023	0.80214	0.01079	-
wheat	1.7e-12	2.1e-13	0.00019	0.13729	2.1e-07	0.02237	4.7e-12	0.00012	1.5e-08

Reproductive output [mg*female^{-1}] Dunn's posthoc test (false discovery rate corrected):

	blood	chlor	hemp	yeast	bone	lupine	fungi	pollen	spiru
chlor	0.08950	-	-	-	-	-	-	-	-
hemp	2.7e-06	0.00053	-	-	-	-	-	-	-
yeast	1.6e-08	4.9e-06	0.30856	-	-	-	-	-	-
bone	0.00053	0.04615	0.15886	0.01367	-	-	-	-	-
lupine	1.8e-09	6.0e-07	0.11082	0.56299	0.00291	-	-	-	-
fungi	0.09792	0.98146	0.00068	7.7e-06	0.04688	9.8e-07	-	-	-
pollen	7.8e-05	0.01153	0.32510	0.04289	0.65668	0.01005	0.01291	-	-
spiru	0.15886	0.87473	0.00102	2.0e-05	0.04688	3.0e-06	0.87497	0.01367	-
wheat	2.0e-12	4.5e-10	0.00546	0.07453	2.0e-05	0.26438	8.8e-10	9.3e-05	6.5e-09
